# Bayes factor benefits for clinical psychology: review of child and adolescent evidence base

**DOI:** 10.12688/f1000research.76842.2

**Published:** 2022-09-23

**Authors:** Thomas B. Bertelsen, Asle Hoffart, Sondre Sverd Rekdal, Rune Zahl-Olsen

**Affiliations:** 1 Department of Clinical Child and Adolescent Psychology, Faculty of Psychology, University of Bergen, Bergen, Norway; 2 Department of Child and Adolescent Mental Health, Sørlandet sykehus, Kristiansand, Agder, 4615, Norway; 3 Research institute of Modum Bad psychiatric hospital, Vikersund, Norway; 4 Department of Psychology, University of Oslo, Oslo, Norway

**Keywords:** evidence-based, Bayesian, Bayes Factor, clinical psychology, child and adolescent psychology

## Abstract

***Background:*** Statistical methods are a cornerstone of research in clinical psychology and are used in clinical trials and reviews to determine the best available evidence. The most widespread statistical framework, frequentist statistics, is often misunderstood and misused. Even when properly applied, this framework can lead to erroneous conclusions and unnecessarily prolonged trials. The implications for clinical psychology are difficulties in interpreting best available evidence and unnecessarily costly and burdensome research. An alternative framework, Bayesian statistics, is proposed as a solution to several issues with current practice. ***Methods:*** Statistical tests of primary outcome measures were extracted from 272 studies, which were cited in 11 recent reviews in the Evidence-based updates series in the Journal of Clinical Child and Adolescent Psychology. The extracted tests were examined regarding relevant features and re-analyzed using Bayes Factors. ***Results:*** When statistical tests were significant, the majority (98%) of re-analyzed tests agreed with such claims. When statistical tests were nonsignificant almost half (43%) of re-analyzed tests disagreed with such claims. Equally important for clinical research, an average of 13% fewer participants per study would have been required if the studies had used Bayes Factors. ***Conclusions:*** Bayes Factors offer benefits for research in clinical psychology through intuitive interpretations, and less costly trials.

## Introduction

Statistical methods are a cornerstone of research in clinical psychology and play an important role when assessing the evidence base of treatments. Such methods are intended to rigorously test posited hypotheses and inform researchers and clinicians whether a treatment is effective or not, why it is effective, and how to improve treatment. In clinical psychology, the importance of appropriate use of statistical methods has been formalized into criteria, which are necessary to evaluate the evidence base for a treatment ( Chambless & Hollon, 1998; Silverman & Hinshaw, 2008). Through the assessment of evidence-based treatments, statistical methods thus have far-reaching impact on what research is further developed, and ultimately what treatment clients receive.

However, researchers in the field of clinical psychology over-rely on a single set of methods, despite certain limitations of these. The vast majority of research within this filed is namely based on the *frequentist* statistical methods, typically the *p*-value and confidence intervals ( Nuijten *et al.*, 2016). Considering the popularity of these methods, it is problematic that they can easily be misinterpreted and lead to challenges in conducting and interpreting studies. When designing a study and using frequentist statistics, one must consider that the reliability of tests is affected by how many tests are performed ( Greenland *et al.*, 2016). As a consequence frequentist methods do not easily allow for monitoring data while it is gathered ( Dienes & Mclatchie, 2018). Moreover, ascertaining reliability requires larger samples and introduce ethical issues when planning and executing a study. The researcher faces a dilemma between gathering enough data to make valid inferences and burdening many clients with research procedures as well as risking delivery of ineffective or potentially harmful treatment to more subjects than necessary. The challenges in interpreting findings based on frequentist methods are related to how these methods work over many repeated trials, and thus the individual trial should be interpreted cautiously ( Greenland *et al.*, 2016). The described challenges are important as clinical decisions on which treatment to deliver are based on such research (for a review see Bakker *et al.*, 2012; Kruschke & Liddell, 2018; Simmons *et al.*, 2011). More particularly, nonsignificant findings (e.g., *p* = .07) may be taken to indicate a lack of effect, when they more appropriately should be taken to indicate an uncertainty about the existence of an effect. This in turn may lead to premature discontinuation of further research and recommendations for practice that overlook potentially effective — but as yet uncertain — interventions.

A promising solution to these issues is Bayesian statistics ( Kruschke & Liddell, 2018). These methods have not been widely applied in clinical psychology, although they have been gaining interest ( Oldehinkel, 2016; van de Schoot *et al.*, 2017). Previous studies have evaluated how *p*-values have been misreported in the field of psychology in general and how Bayesian methods can be beneficial for evaluating evidence-based treatments in an adult population ( Sakaluk *et al.*, 2019; Williams *et al.*, 2020). However, the clinical consequences in terms of potentially increased sample sizes and recommendations for practice have not been investigated. In this article, we motivate researchers in clinical psychology to adopt Bayesian statistics by describing and empirically investigating the practical benefits of using Bayes Factors compared to *p*-values. To accomplish this, we conducted a reanalysis of studies included in 11 recent evidence base updates on treatments published in the Journal of Clinical Child and Adolescent Psychology (JCCAP) to investigate how the current practice of frequentist statistics affects the conclusions that are reached in this field of research and how the field can benefit from adopting Bayes Factors in place of or in addition to existing methods. The overall aim is to assess the clinical consequences of employing Bayes Factors versus *p-*values in research.

### Frequentist and Bayesian methods

To enter a discussion of the clinical consequences of statistical choices, preliminary clarification of frequentist and Bayesian methods is needed. The frequentist methods most widely used in clinical psychology today have their origin in the *p*-value developed by Ronald Fisher (1925). This represents the probability of observing the treatment effect one has observed (or larger treatment effects) if there is no treatment effect. The hypothesis of no treatment effect is *H*_0_. If *H*_0_ is true, the *p*-value will vary between 0 and 1, with equal probability of any value ( Rouder *et al.*, 2009). That is, if *H*_0_ is true, we are equally likely to observe a *p* of .01 and .56. If *H*_0_ is false, *p*-values will tend to be small. The *p*-value informs us of *H*_0_, the hypothesis of no treatment effect, but it cannot directly tell us anything about the alternative hypothesis ( *H*_1_), that the treatment is effective.

To improve this situation, Neyman and Pearson (1933) elaborated on the *p*-value method. They proposed a decision rule for concluding that the treatment works or not, after conducting *several independent studies* ( Berger, 2003; Christensen, 2005). This decision rule does not inform the researcher whether she has made the correct decision for each study individually, but she would know how often she would make the wrong decision if the experiment was repeated indefinitely ( Dienes, 2011; Szucs & Ioannidis, 2017). The decision rule is based on deciding, before the trial, which specific effect-size value is expected if there is no treatment effect ( *H*_0_), and which if there is a treatment effect ( *H*_1_). One then decides on acceptable values for α and β. The measure α is the error rate, which is the proportion of times the researcher will be wrong in concluding the treatment *does work* if the experiment was repeated indefinitely. The measure β is the proportion of times the researcher would be wrong in concluding the treatment *does not work* if the experiment was repeated indefinitely. Based on the values of *H*_0_, *H*_1_, α, and β, one computes a sample size. If the researcher has followed this method carefully, she can choose to conclude that the treatment does work if the *p*-value is lower than the set α-level or choose to say the treatment does not work if the *p*-value is above the set α-level ( Berger, 2003; Szucs & Ioannidis, 2017). The α-level and *p*-value criteria are typically set to .05 based on a rule of thumb introduced by Fisher (1925)

Bayesian methods are an alternative to frequentist. These are based on the theorem by Thomas Bayes (Bayes, 1763), which states that the conditional relationship between two variables A and B is defined by P(A|B) = [P(B|A)*P(A)]/P(B). The *Bayes Factor* ( *BF)* is an essential aspect of this approach as it formalizes how the data inform us. The *BF* indicates the relative strength of evidence in the data for two competing theories ( Dienes, 2014). When noted as *BF _10_* the Bayes Factor denotes the ratio of evidence for the alternative hypothesis compared to the null hypothesis. An example could be comparing the theory of a treatment effect to the theory of no treatment effect. The *BF _10_* varies between 0 and ∞, where 1 indicates that the data does not favor one hypothesis over the other. Values above 1 indicate evidence for the alternative hypothesis, whereas values below indicate evidence for the null hypothesis. The *BF* is considered a degree of evidence and scales with the number of observations confirming one theory over the other. Since the *BF* gives us degrees of evidence *and* considers two competing hypotheses, it can give us three possible conclusions based on a single experiment, depending on the *BF*s magnitude: (a) There is evidence of a treatment effect, (b) There is evidence of no treatment effect, and (c) There is equal evidence for there being an effect and there not being an effect ( Dienes & Mclatchie, 2018). There are no set rules for what counts as strong evidence and what does not, and one must consider the gravity of decisions when deciding what constitutes the strength of evidence. In a life-or-death situation 25:1 odds that a treatment works may not suffice, whereas when deciding what day of the week to begin a therapy session, a 2:1 odds for a preferred day may be more than enough to base a decision on.

### Clinical implications

The difference between frequentist and Bayesian methods is important when considering how many participants to include in clinical trials, and when to stop recruiting. Particularly the praxis of conducting statistical tests before a study is finished (hereafter monitoring data) is treated differently by the frequentist and Bayesian methods. Because the *p*-value is equally distributed between 0 and 1 when there is no treatment effect ( Rouder *et al.*, 2009), some *p*-values will be below .05 and some will be above, even when there is no treatment effect. Thus, researchers will always be able to obtain *p* < .05 if they conduct enough tests. When performing one test and using *p* < .05 as a decision criterion, the error rate is 5%, but when performing 20 tests and using the same decision criterion the error rate increases to 25% ( Armitage & Rowe, 1969). To prevent this and to keep the error rate stable, one must correct the *p*-value for the number of tests performed ( Kruschke & Liddell, 2018). Thus, frequentist methods are penalized by the number of tests performed. As a consequence, researchers face an ethical dilemma with regard to the trade-off between sample size and ability to monitor data as it is collected. If the treatment is effective and not harmful, it would be unwise to monitor subjects often because this would lead to the unnecessary prolonging of the trial and more people receiving an inferior control treatment. However, if the treatment is not effective or even harmful, it would be unwise to monitor subjects infrequently. This would lead to more people receiving harmful or inferior treatment.

Bayesian statistics offer a solution to this dilemma, by questioning why it should matter how many, or when, tests are performed ( Dienes, 2011). In frequentist methods, one assumes that *H _0_* is true and then proceeds to describe the observed data with probability statements. The question asked is “what is the probability of observing these data, if there is no effect?”. In Bayesian statistics, one assigns probability statements to the hypothesis not the data. The question asked here is “What is the probability that there is a treatment effect when we have observed these data?”. Because of this difference in the view of the data, Bayesian analysis can be performed as many times as you want without jeopardizing the reliability of the results. You can stop sampling when results are convincing either way. Bayesian methods are affected by the how convincing the data are of a hypothesis and how much evidence one requires to make a satisfactory conclusion ( Dienes & Mclatchie, 2018; Dienes, 2011; Wagenmakers *et al.*, 2018).

Since the *BF* does not depend on the testing plan of the researcher, it does not matter if researchers test hypotheses only at the end of a clinical trial or if one tests every time a new subject enters. Bayesian methods allow gradual decisions to be made based on continuous monitoring of data because the *BF* is not affected by testing intentions. The implication of this is that Bayesian methods may reach conclusions in clinical trials faster than classical methods. Thus, using the *BF* may pose less burden on participants.

In addition to the speed of trials, Bayesian methods, such as *BF*s offer advantages in terms of interpreting findings. After a trial, researchers wish to know whether the treatment works or not, that is, whether *H*_1_ or *H*_0_ is true. Such questions are however not easily answered using frequentist methods. The *p*-value assumes that *H*_0_ is true and gives the probability of our observation under that assumption. Thus, the *p*-value cannot be used to indicate whether a treatment works or not and is at best a weak heuristic for decision making. The method proposed by Neyman and Pearson does inform us whether the treatment works or not, however, only after many repeated trials. Indeed, using this method, we must accept that the single trial does not inform us.

Contrary to the frequentist methods, the *BF* allows us to make inferences based on each conducted study, with higher values indicating stronger support for the theory that there is a treatment effect. The *BF* also allow us to gather evidence for the theory that there is no treatment effect. This is underappreciated but highly advantageous for clinical research. Just as important as it is to know that a treatment works, it is equally important to know that it does not work and that it should be aborted. Another unique benefit of *BF*s is that they allow us to distinguish between evidence for a theory and lack of evidence ( Gallistel, 2009; Morey *et al.*, 2016; Wagenmakers *et al.*, 2018). Imagine a researcher expecting a 40% rate of remission if there is no treatment effect (from pure placebo) and an 80% rate of remission if there is a treatment effect. If she in a study observes a 60% rate of remission, this will not seem to favor one theory over the other and the *BF* will inform the researcher about this. The approach by Fisher may conclude that the observed effect is improbable if there is no treatment effect. With several equal results, the method by Neyman-Pearson leads the researcher to reject the theory of no effect. Thus, data may lead to conclusions in the frequentist methods when the data does not suggest that one theory is more likely than the other. This difference between the statistical paradigms is of importance for clinical researchers since they will lead to different motivations for further research, and different recommendations for practicing clinicians. Non-significant findings may potentially lead to reduced interest in a research area and recommendations against using such interventions in practice. This is problematic if the *BF* suggest that the treatment may be effective.

### The present study

We have described some major benefits of the Bayesian statistics, with emphasis on *BF*s, compared to frequentist approaches and highlighted the practical implications of these. To better understand the real-world implications of *BF*s versus frequentist we aim to empirically demonstrate how a specific subfield of clinical psychology has been affected by statistical choices. To investigate this, we selected the meta-analytic reviews in the evidence base updates on treatments published in the *Journal of Clinical Child and Adolescent Psychology* (JCCAP) between 2017 and 2019. From these we extracted the statistical tests on primary outcome measures, and reanalyzed these using *BF*s. The subfield of clinical child and adolescent psychology was selected for two reasons. First, within this subfield there is an inherent power imbalance between adult researchers and non-adult clients. Thus, it is of particular importance to follow the ethical imperative to not burden clients through research. Second, the evidence-base updates series in JCCAP has highly transparent reporting standards, allowing for easy identification of individual studies and primary outcomes within studies. We investigated two primary hypotheses:

1. The conclusions reached in current practice would have been reached with a smaller sample size if data had been monitored using Bayes Factors.
2. Current research practice indicates an effect or lack of effect when there is no evidence in the data for such conclusions.

## Methods

### Study design

The study is a cross-sectional analysis of a strategic sample of articles that laid the foundation for the evidence base in child and adolescent clinical psychology between 2017–2019. The sample was selected to investigate what statistical analyses make up the evidence base in clinical child and adolescent psychology. The definition of evidence base was articles contained within the evidence base update series of Journal of Child and Adolescent Clinical Psychology. Thus it is important to note that our sample does not contain the entire evidence base for child and adolescent psychology. Rather it contains those articles that we believe have had substantial influence as evidence base.

### Inclusion and exclusion criteria

A set of 11 meta-studies, reporting on treatment effects, in the evidence base updates series of the Journal of Child and Adolescent Clinical Psychology published between 2017–2019 was selected to extract individual articles from. These include the evidence base updates on psychosocial treatments for: early childhood anxiety and related problems ( Comer *et al.*, 2019), children and adolescents exposed to traumatic events ( Dorsey *et al.*, 2017), children and adolescents with attention-deficit/hyperactivity disorder ( Evans *et al.*, 2014), pediatric obsessive-compulsive disorder ( Freeman *et al.*, 2018), self-injurious thoughts and behaviors in youth ( Glenn *et al.*, 2019), disruptive behaviors in children ( Kaminski & Claussen, 2017), ethnic minority youth ( Pina *et al.*, 2019), child and adolescent depression ( Weersing *et al.*, 2017), and pediatric elimination disorders ( Shepard *et al.*, 2017) as well as outpatient behavioral treatments for adolescent substance use ( Hogue *et al.*, 2018) and treatments for youths who engage in illegal sexual behaviors ( Dopp *et al.*, 2017). Inclusion criteria for individual articles within meta-studies were the presence of a control group condition, the description of quantitative measures of treatment outcomes that allowed the calculation of Cohens *d* ( Cohen, 1988) and Bayes factors from summary statistics. Summary statistics eligible for inclusion were contingency tables, regression models, generalized linear regression models, binary proportions, χ ^2^ values, *F*-values, means, and *SD* or *SE*, *t*-tests, correlations, odds ratios, hedge’s *g*, and η ^2^. The selection of studies was based on a full reading to assess eligibility. Study selection was conducted independently by the first and third authors on the evidence base update on early anxiety interventions ( Comer *et al.*, 2019), with complete agreement on which studies to include. Further information about which studies were included can be found in the extended data ( Bertelsen, 2022).

### Data collection

Measures associated with primary outcomes within each study were extracted by hand. These included the effect size measure, how authors interpreted the *p*-value, and the *n* associated with the test. If multiple summary statistics were available for the same treatment outcome (e.g., reporting *t*-tests and means and standard deviations), the summary statistics that required the least assumptions about the data were preferred over those that required more assumptions. That is, means and standard deviations were preferred over *t*-values, which were preferred over *F*-values, which were preferred over regression models, and so on. A subset of 29 (11% of included) articles was independently assessed by the first and third authors, with complete agreement on the description and interpretation of *p*-values, and which effects were primary. For the effect-size measure and *n* associated with it, there was high inter-rater reliability (Cronbach’s α = 0.91). Disagreement between authors was handled through discussion.

An outcome was defined as primary if described as such within the article. If such information was not available, the definition was based on whether the evidence base update article, in which it was included, had treated it as a primary outcome. If this was not available, it was based on whether other studies within the same field had defined the present outcome as primary.

Assessment of whether a test was treated as significant or not was based on authors’ reports. If such reports were not available, tests were supposed to be treated as significant based on the described cut-offs for significance elsewhere in the article. If this was not available, the cut-off of *p* < .05 was used.

The authors' interpretation of the *p*-value was coded as either indicating an effect of treatment, no effect of treatment, or a negative effect of treatment (e.g., the control group performs better). If authors described a significant treatment effect or described that the treatment was better or outperformed the control condition, this was taken as indicating a treatment effect. If authors reported no differences, not significantly different, or simply did not describe any outcome measure, this was taken as indicating equality. If authors described a treatment as worse than, or having a negative effect, or control groups being superior, this was taken to indicate a negative effect of treatment.

The *n* associated with a test was extracted for participants in the treatment condition and the control condition. If Intent-To-Treat analyses were available, these were used and the *n* associated with these was extracted.

Calculation of Bayes factors from summary statistics was conducted in R ( R Core team, 2020) using the BayesFactor package ( Morey & Rouder, 2018). The aim of these methods is to calculate the Bayes Factor as a comparison between the likelihood of the null-model and the alternative using priors that have been developed to be sensitive to change, while becoming increasingly stable as sample size increases. These methods are described by Rouder *et al.* (2009) for outcomes that allowed the calculation of *t*-statistics (81% of summary statistics). For outcomes for which binomial proportions were available (13% of summary statistics), or computable, Bayes factors were calculated using the method described by Kass and Vaidyanathan (1992). In the case of χ ^2^-statistics (5% of summary statistics), the method described by Johnson (2008) was used to calculate Bayes factors. In the cases of tests, in which only *R* ^2^ values were available (0.4%), the method described by Rouder and Morey (2012) was applied to calculate Bayes factors. Finally, some outcomes only gave results as odds ratios (0.4% of summary statistics), without other available information. These outcomes were recalculated into Cohens *d* and interpreted as *t*-statistics and Bayes factors were calculated based on this.

### Statistical analysis

In the presented analyses, the inferential statistic is either represented as the Bayes factor ( *BF*) or credibility interval. The *BF* describes the hypothesis of an effect ( *H*_1_) over the hypothesis of no effect ( *H*_0_) unless otherwise specified. The credibility interval (CI) describes the interval where the parameter is with 95% probability, with values closer to the centre being more probable. For all analyses, priors were specified with the intent that conclusions should be primarily influenced by observed data. For continuous outcomes the prior distribution was Cauchy distribution with a location parameter of 0 and a scale of 0.7. For categorical outcomes the prior was a beta distribution with parameter a = 1 and b = 1.

***Analysis of p-value reporting***

To ensure that differences between *BFs* and *p-*values did not reflect erroneously reported *p-*values, the sample was checked for inconsistencies in reported statistics using the *p*-checker app ( Schönbrodt, 2018). Tests that were reported as significant or nonsignificant at a set level but reported a statistic that would lead to a *p*-value in the opposite direction were coded as *mismatched.* Such situations would be when *p* < .05 was reported, but the test statistic resulted in *p* > .05 or vice versa. This implies that either the summary statistics, the reported *p*-value, or the assumptions underlying the tests were incorrect. After removing tests that indicated mismatched *p*-values, we calculated *BF*s based on the reported statistics. This was done to ensure that differences between *BF*s and *p*-values did not reflect erroneously reported *p*-values.

***Sample size with Bayes Factors***

To analyze the expected required *n* if Bayes Factors had been used in the studies, we used the BFDA package in R ( R Core Team, 2020; Schönbrodt & Wagenmakers, 2018), to simulate 10,000 repeated studies based on the median observed effect size of the articles. We simulated a design where data was monitored continuously with stopping rules being either (a) achieving a *BF* > 9 or (b) achieving a sample size that would have 95% power to achieve a *BF* > 3 if the effect was present. In performing these simulations, we assumed a non-informative prior. The first stopping rule ( *BF* > 9) was chosen based on preliminary testing suggesting this stopping rule would result in a false-positive rate below .05, which we believe would be reasonable to researchers accustomed to using α = .05. The second stopping rule ( *BF* > 3, with 95% power) was chosen as a futility limit, based on our belief that researchers would not sample indefinitely if the data gave them no indication of an effect and they would expect to see an indication if there was an effect.

***Categorization of Bayes factors***

To exemplify the difference between inference based on *p*-values and *BF* we divided the *BF*s into eight categories based on recommendations in common use ( Wagenmakers *et al.*, 2018). We describe *BF*s between 0.33 and 3 as “not worth a bare mention”, between 0.05–0.33 and 3–20 as moderate evidence for *H*_0_ and *H*_1_, respectively. *BF*s between 0.0066–0.05 and between 20–150 are seen as strong evidence for *H*_0_ and *H*_1,_ respectively, whereas values below 0.0066 and above 150 are seen as very strong evidence for *H*_0_ and *H*_1,_ respectively. These categorizations should not be seen as objective demarcations of truth, but rather a simplified model for the reader. In our analysis we mainly focus on whether a *BF* is above or below 1 and whether it is within the range of “not worth a bare mention” (0.33–3). The aim of this focus is to assess how results would have been interpreted differently using *BFs* both in terms of direction of conclusion and whether evidence was strong enough to draw a conclusion.

## Results

In the 11 evidence base updates assessed, 309 articles were described. Of these, we were unable to obtain 5 of the original articles. Additionally, 12 articles did not report summary statistics that allowed for the recalculation of Bayes factors. Finally, 5 articles did not include a control group or comparison condition (i.e. pre-treatment measures or benchmark) and were thus not selected for further evaluation. In the remaining 287 articles, 24 appeared in more than one evidence base update. In the final 263 articles, 272 studies were included for analysis, with 26170 participants ( *M* = 96.5, *Mdn* = 65, min = 6, max = 832), and 2431 statistical tests were extracted, with a mean of 9.4 statistical tests on primary outcome measures per study (min = 1, max = 175). The mean observed power of the studies was 0.77 (min = .03, max = .99). Of the 2431 statistical tests, 171 showed indication of mismatched *p-*values and thus 2260 were used for further analysis. See Table 1 for a summary of descriptive statistics.

**Table 1.** Descriptive statistics for studies.

	*p*-value mismatch ^a^	No. of tests	*N*	Tests/ *N*	Observed power	Observed median effect size	Mean *BF ^b^*
Mean	0.63	9.35	96.48	0.18	0.77	0.66	7.60e+77
Median	0	6	65	0.09	0.96	0.47	197.64
Min	0	1	6	0.01	0.03	0.01	0.14
Max	76	175	832	3.46	0.99	12.00	2.06e+80
Total	171	2431	26170	-	-	-	-

*Note.* Summary statistics describe mean, median, minimum and maximum per included article.

^a^ A *p*-value mismatch indicates a mismatch between *p*-values calculated from summary statistics and those reported, that would have lead to a difference in interpretation.

^b^ The Bayes Factor ( *BF*) indicates the ratio of evidence for an effect compared to evidence against an effect.

### Consequences of statistical choices

**Figure 1.**
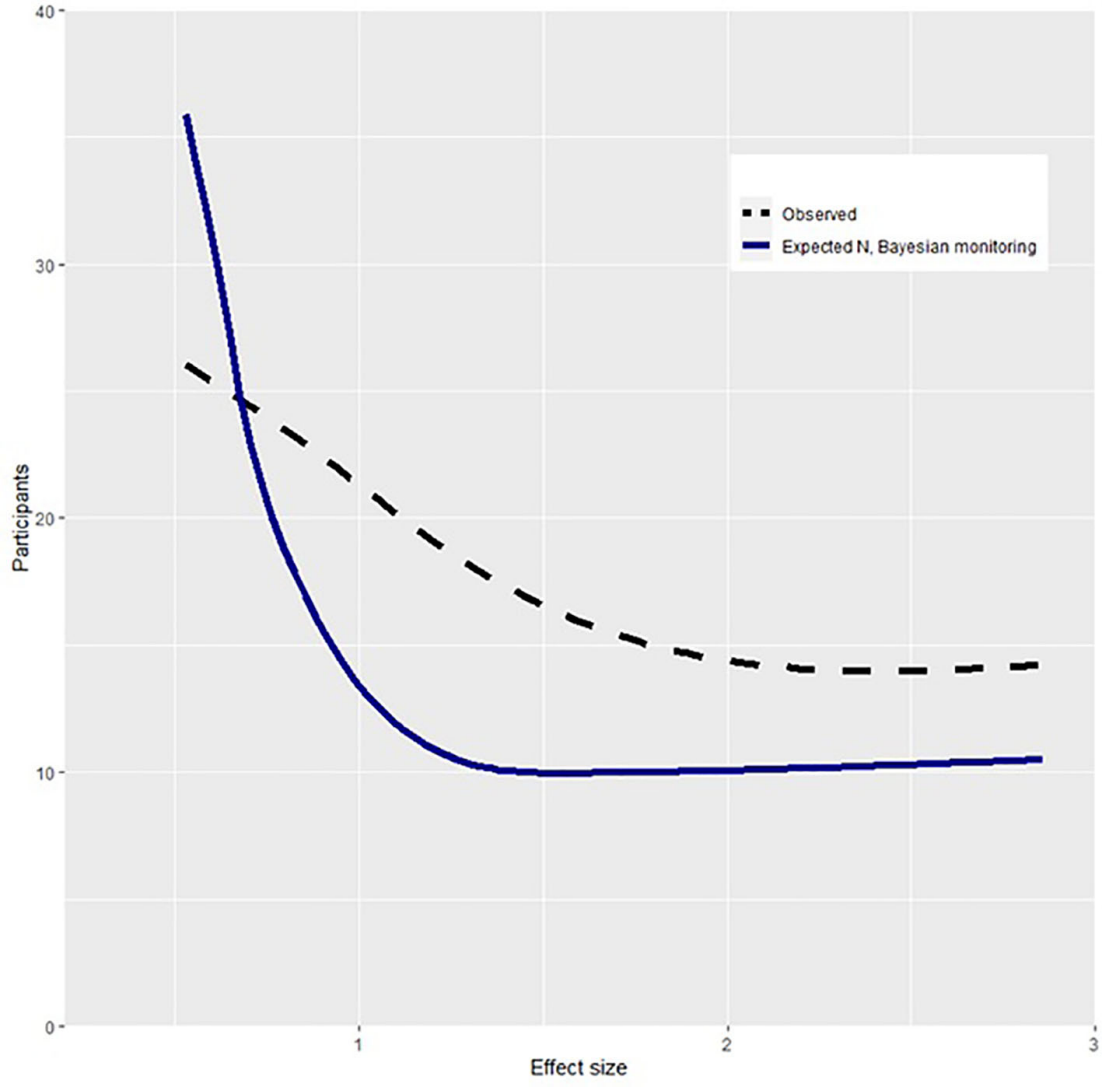
Participants required in Bayesian and observed design as a function of effect size. *Note.* Effect size is measured in Cohens *d.* Participants describes number of observed or required participants per group. The required N with Bayesian monitoring describes the expected participants per group if one monitored data and stopped collecting when reaching a Bayes Factor of > 9.

Our first hypothesis was that conclusions reached in current practice would have been reached with a smaller sample size if data had been monitored using Bayes Factors. To assess this, simulated replications of each study were conducted based on the median observed effect size. The observed sample size and that required by Bayesian methods are shown in Figure 1. This shows that the Bayesian method requires smaller sample sizes than the observed studies as a function of effect size. Using Bayes Factors and monitoring data until reaching a *BF* > 9, studies would have reached conclusions with an average of 10.4 (95% CI [4.8, 16.1]) fewer participants per control group compared to what was observed. The average relative decrease in sample size was 12.7% (95% CI [5.0, 20.5]). Across studies, 1656 (95% CI [1649, 1664]) fewer participants would have been required if Bayes Factors had been applied. To understand the implications of a smaller required sample, a subset of studies that reported on suicide attempts ( *k* studies = 17) were further analyzed. Within the Bayesian replication, using power equal to the described studies, an estimated 67 individuals who attempted suicide would not have been placed in a control group due to fewer required participants. Overall, these findings support the hypothesis that smaller sample sizes would be required if monitoring data using Bayes Factors.

### Inference based on p-values and Bayes factors

Our second hypothesis was that current research practice indicates an effect or lack of effect when there is no evidence in the data for such claims. To assess this hypothesis, *BF*s were calculated on the basis of summary statistics and compared to the claims made in the articles. Results shown in Table 2 indicate that when findings were claimed to be support for *H*_1_ the majority of *BF*s were in agreement with that claim. Of the *p*-values deemed significant, only 1.8% ( *n* = 21) were evidence in the direction of *H*_0_ ( *BF*_10_ < 1), whereas 3.3% ( *n* = 39) were “not worth a bare mention”. In the case of findings claimed to be support for *H*_0_, the *BF*s did not uniformly support such claims. Of the *p*-values deemed nonsignificant 43.6% ( *n* = 470) were evidence in the direction of *H*_1_ ( *BF*_10_ > 1) and 50% ( *n* = 539) were “not worth a bare mention”. Among the tests that were reported as nonsignificant, but the *BF* indicated evidence in the direction of *H*_1_, 61.3% ( *n* = 301) had used correction methods to lower the α-level. These findings indicate that when research indicates the presence of an effect there is evidence in the data for such a claim. However, these findings also suggest that when research indicates a lack of an effect the evidence in the data does not generally support such a claim.

**Table 2.** Comparison of Bayes Factors to significant and nonsignificant findings.

	*BF* evidence for *H*_0_				*BF* evidence for *H*_1_			
	Very strong ( *BF* < 0.0066)	Strong ( *BF* [0.0066-0.05])	Moderate ( *BF* [0.05-.333])	Not worth a mention ( *BF* [0.333-1])	Not worth a mention ( *BF* [1-3])	Moderate ( *BF* [3-20])	Strong ( *BF* [20-150])	Very strong ( *BF* < 150)
Inferential tests								
By reported *p*-value								
*p*-value reported as evidence for *H*_0_	0	0	244	365	174	131	87	78
*p*-value reported as evidence for *H*_1_	0	0	10	11	28	41	106	985
*By direction of argument*								
*Treatment*	0	0	12	19	34	52	116	978
*Equality*	0	0	241	355	165	120	74	73
*Control*	0	0	1	2	3	1	3	11

*Note.* All Bayes Factors ( *BF*) describe evidence for *H*_1_ relative to evidence for *H*_0_ *. BF*s above 1 indicate increasing evidence for *H*_1_, whereas *BF*s below 1 indicate increasing evidence for *H*_0 *.*_ The subset of test by direction of argument describes *BF*s of tests that were argued to indicate an effect of treatment (Treatment), an equality between control and treatment or no effect (Equality), and a superior effect of control group or iatrogenic effect of treatment (Control).

To expand on these findings Table 2 depicts *BF*s in relation to what the authors of articles argued the test to indicate. When authors had argued for the existence of a treatment effect 94.6% of tests indicated support for this statement ( *BF* > 3), whereas 4.3% indicated not much evidence ( *BF:* 0.33–3) and 1.9% indicated evidence for lack of an effect ( *BF <* 0.33). When authors had argued that a test indicated a lack of difference or an equality 23.4% of tests supported this statement, whereas 50.5% indicated not much evidence and 26.1% indicated evidence for the existence of an effect. When authors had argued that a test indicated superiority of the control group condition 71.4% of tests supported this statement, whereas 23.8% indicated not much evidence and 4.8% indicated evidence for the lack of superiority of control group condition.

## Discussion

The purpose of this paper was to investigate how the widespread use of frequentist statistics affects research in clinical psychology, and what benefits the field may have from incorporating Bayesian methods, such as Bayes Factors. 11 recent evidence base updates were selected, and 2431 tests were extracted from 272 included studies. Sample size estimation with Bayesian methods using data-monitoring indicated that we would expect a decrease in the expected sample size per study of 12.7% due to being able to monitor the data while it was gathered. Among tests in which *p*-values were reported correctly, 55% of the tests did not strongly support the hypothesis claimed by the *p*-value when reanalyzed with Bayes Factors. Unfortunately, 22% of tests supported the opposite hypothesis of that stated by the *p*-value. Of clinical importance when statistical tests were used to argue for a lack of effect, only 23% of tests were in agreement with this, with slightly more (27%) indicating the existence of an effect.

### Designing and conducting studies

The results indicate that the required sample size in studies could have been considerably reduced had Bayes Factors been adopted. This is due to the ability of these methods to allow for continuous monitoring of data and altering of the trial as it is in progress. These aims are difficult to attain with corresponding frequentist methods, which require a large and predefined sample size to allow for multiple comparisons ( Berry, 2004). The ethical importance of requiring lower *n* is particularly poignant in the case when negative outcomes may befall participants in control group conditions. In the current study, it was found that 67 individuals who attempted suicide and were placed in a control group would not have been placed in a control group if Bayes Factors were applied. Although this does not directly support the statement that they would not have attempted suicide if Bayes Factors had been applied, one cannot preclude that allocation to the control instead of the treatment group contributed to demoralization and suicidality.

### Interpretation of inferential statistics

Overall *p*-values described as significant indicated evidence in favor of *H*_1_ when reanalyzed with *BF*, with 96% ( *n* = 1132) having a *BF* above 3. Of greater concern is the finding that only 23% ( *n* = 244) of the nonsignificant findings showed evidence in the direction of *H*_0_ ( *BF*_10_ < 0.33), with 27% ( *n* = 296) showing evidence in favor of *H*_1_ ( *BF*_10_ > 3) and 50% ( *n* = 539) showing not much evidence at all ( *BF*_10_ 0.33-3). These findings are surprising, and are the opposite of what is usually raised as a critique of frequentist methods ( Colquhoun, 2014). A substantive explanation for this is the low power of studies or using error-control procedures that lowered power. In other words, the studies analysed had low power to detect an effect and set a threshold that was to restrictive to observe an effect. In some ways this may be seen as positive, and as an attempt to avoid false positives ( Mayo, 2018). However, from a clinical perspective false negative are equally important seeing as negative findings in clinical psychology may reduce research and funding available. This is the case at least for the analysed studies in this paper, where null findings are associated with fewer studies and abandonment of research on certain clinical interventions.

Whether we are using frequentist or Bayesian methods, we want to make correct conclusions. However, Bayesian methods and frequentist methods may reach different conclusions, due to different assumptions and thresholds. This may at first glance seem confusing and leave researchers asking what to believe – the *BF* or the *p*-value? Such phenomena have been described as “Lindley's paradox” ( Shafer, 1982), but do not constitute any real paradox. Instead, these showcase the difference in interpretation of data in the frequentist framework and the Bayesian. There is no paradox between (a) the frequentist interpretation: observing this data is not unlikely if *H _0_* is true and (b) the Bayesian interpretation: *H*_1_ is more probable than *H _0_* if we observe this data. Thus, Bayesian methods cannot be said to be more true than frequentist methods and conclusions in both frameworks depend on thresholds chosen. However, a distinct benefit of Bayesian methods from a clinical perspective is the ability to withhold judgement by allowing to reach the conclusion of no conclusion (e.g., *BF*s between 0.33-3 in this study).

### Strengths and limitations

A strength of the current study is that it evaluates the performance of frequentist methods and Bayesian methods on findings that are clinically meaningful. Previous studies have assessed *p*-values and *BF*s based on summary statistics regardless of whether these were supplementary (e.g., correlation tables or sample demographics) or of primary concern (clinical outcomes) (e.g., Nuijten *et al.*, 2016; Wetzels *et al.*, 2011). Other studies have evaluated the use of Bayesian statistics and their benefits for clinical practice from a theoretical perspective or using simulation studies ( Dienes & Mclatchie, 2018; Kruschke & Liddell, 2018; Wagenmakers *et al.*, 2018). To our knowledge, this is the first comprehensive empirical assessment of how frequentist compared to Bayesian methods affect the field of clinical psychology.

Some limitations of the current study are important to note. The effect-measures and Bayes factors calculated in the present study are based on summary statistics from published articles and not on the observed data. Regarding the calculation of effect size measures and *BF*s, this should be of little consequence, given that the margin of error between summary statistics and observed data are generally inconsequential ( Lin & Zeng, 2010).

A second limitation of the current study relates to estimates of the required sample size if one had used Bayesian methods. These estimates are based on simulated replications of studies and not actual studies and thus only describe what we would *expect* to see if we repeated the studies, not what we necessarily would observe. A central aspect of this limitation is that simulations are based on the case where *H*_1_ is defined as true. However, the intent of this paper is not to test whether the findings from the original papers are correct or not, but rather showcase how the pragmatic approach using Bayesian methods could benefit research in clinical psychology. In line with this, it is not possible to know whether fewer suicide attempts would have been made if a Bayesian design was employed. However, we can make the counterfactual argument that these suicide attempts were seen in control groups given the observed design, and we would not have expected them given the use of Bayesian methods.

Another possible limitation of the current study is the use of non-informed priors, that is, assuming that there was no pre-existent evidence for or against the hypotheses. One may rightfully raise the question that the findings would have been different had we used alternative, more informed, priors. This is correct, and it is important to acknowledge that the findings in this article are not intended to absolutely refute the findings of the analyzed articles. The intent is rather to question whether statistics are best represented as a dichotomous choice, as in frequentist methods, or as degrees of evidence, as in Bayesian methods. Indeed, the ability to specify priors is itself helpful for research as it allows a fruitful discussion of how knowledge accumulates. Specification of priors is further useful as they may increase the power of studies by incorporating information from previous studies or clinical experience. Additionally, Bayesian priors represent a more reasonable assumption than that of the *p*-value, which assumes that *H*_0_ is true. It seems odd that one would wish to carry out a study on the effect of a treatment if one from the onset assumes that there is no effect.

### Clinical implications

The presented findings have implications for current and future evidence-based clinical practice. Currently evidence-based practice is defined as the integration of best available research with clinical expertise ( APA Presidential Task Force on Evidence-Based Practice, 2006). However, it is not specified how to integrate these two. Without systematic integration, meta-studies such as the evidence-based update series may be prioritized over sound clinical expertise. This is of particular concern given that 77% of nonsignificant findings from the evidence-based update series did not indicate a lack of effect. In addition to giving a non-dichotomized description of findings, Bayesian methods also allow a simple heuristic to integrate clinical expertise with research findings. This is done by specifying the *BF* and prior belief based on clinical experience. As an example, a researcher may have considerable experience with hypnotherapy for enuresis leading him to be 90% certain that this is an effective treatment. The researcher learns about a study that found nonsignificant effects of this treatment and calculates the *BF* to be 1.5, thus weak evidence that hypnotherapy is ineffective. His prior odd were 9:1, the *BF* is 1.5:1 which means his posterior odds is 13.5:1, expressed as a probability his posterior belief that the treatment is effective is 93%.

The implications for future evidence-base are related to the possibilities afforded by Bayesian methods due to the ability to continuously monitor data as it is collected and alter treatment while a study is ongoing. In addition to reducing sample size needed this also allows for studying domains that are otherwise difficult due to ethical concerns. An important case is studies of populations where suicide may be a potential outcome. Despite the ethical imperative to improve and find effective treatments for such populations they may be understudied due to ethical issues that are a consequence of frequentist design. Using Bayesian methods one can conduct studies on such population in a way that closer resembles clinical practice, where one continuously tweaks current practice based on available information. Such changes in research opportunities may lead to important discoveries with ramifications for clinical practice.

## Conclusions

Clinical psychology seems to be comprised of researchers who are pragmatic, concerned with study ethics, and who want to know what works for whom. Frequentist methods are at odds with these characteristics: they place researchers in ethical dilemmas while collecting data, and force dichotomized thinking on researchers. Some suggest that researchers should suit their research to accommodate their statistics ( Kerr, 1998); we suggest the opposite – researchers should suit their statistics to accommodate their research. By using Bayesian statistics, many of the practices which are currently problematic for the field (i.e., monitoring data, interpreting findings) will no longer be so. This does not imply that researchers should be careless. However, the type of considerations researchers make may become more clinically meaningful when switching to Bayesian methods.

Researchers should not need to be reluctant to monitor data because this may alter the validity of their inference. Instead, they should assess intervention effects continuously and make appropriate alterations of studies as they are conducted. Researchers should not need to interpret findings in a binary fashion. Rather, they should test competing substantial hypotheses and assess to which degree these are supported.

## Data availability

Open Science Framework: Bayes Factor Benefits for Clinical Psychology. https://doi.org/10.17605/OSF.IO/UB5PJ ( Bertelsen, 2022)

This project contains the following underlying data:

• BayesFactorOutcomeDataShare.csv (Contains data used for analysis of all tests performed, contains information on BFs of individual tests and whether the test was assessed as significant)
• BFSpeedDatashare.csv (Summary data of data used to compare sample size required for Bayesian studies. Contains reported sample size and recalculated sample size as well as Mean cohens d)
• DataKey.docx (Contains data key for variables in BayesFactorOutcomeDatashare.csv and BFSpeedDatashare.csv)
• Supplement.docx (Contains references to all studies included and reanalyzed as well as which meta-analysis they were related to)

Data are available under the terms of the Creative Commons Zero “No rights reserved” data waiver (CC0 1.0 Public domain dedication).

## References

[ref1] APA Presidential Task Force on Evidence-Based Practice: Evidence-based practice in psychology. *Am. Psychol.* 2006;61(4):271–285. 10.1037/0003-066X.61.4.271 16719673

[ref49] Armitage CK Rowe BC : Repeated Significance Tests on Accumulating Data. *J. R. Stat. Soc. Ser. A (General).* 1969;132(2):235–244.

[ref2] Bakker M Dijk A van Wicherts JM : The Rules of the Game Called Psychological Science. *Perspectives on Psychological Science: A Journal of the Association for Psychological Science.* 2012;7(6):543–554. 10.1177/1745691612459060 26168111

[ref3] Berger JO : Could fisher, jeffreys and neyman have agreed on testing?. *Stat. Sci.* 2003;18(1):1–32. 10.1214/ss/1056397485

[ref4] Berry DA : Bayesian Statistics and the Efficiency and Ethics of Clinical Trials. *Stat. Sci.* 2004;19(1):175–187. 10.1214/088342304000000044

[ref5] Bertelsen TB : Bayes Factor Benefits for Clinical Psychology. 2022, January 20. 10.17605/OSF.IO/UB5PJ PMC10558984 37809055

[ref6] Chambless DL Hollon SD : Defining empirically supported therapies. *J. Consult. Clin. Psychol.* 1998;66(1):7–18. 10.1037/0022-006x.66.1.7 9489259

[ref7] Christensen R : Testing fisher, neyman, pearson, and bayes. *Am. Stat.* 2005;59(2):121–126. 10.1198/000313005X20871

[ref8] Cohen J : *Statistical power analysis for the behavioral sciences.* (2nd ed.). Lawrence Erlbaum Associates;1988. 10.4324/9780203771587

[ref51] Colquhoun D : An investigation of the false discovery rate and the misinterpretation of p-values. *R Soc Open Sci.* 2014;1(3):140216. 26064558 10.1098/rsos.140216 PMC4448847

[ref9] Comer JS Hong N Poznanski B : Evidence base update on the treatment of early childhood anxiety and related problems. *J. Clin. Child Adolesc. Psychol.* 2019;48(1):1–15. 10.1080/15374416.2018.1534208 30640522

[ref10] Dienes Z : Bayesian Versus Orthodox Statistics: Which Side Are You On?. *Perspectives on Psychological Science: A Journal of the Association for Psychological Science.* 2011;6(3):274–290. 10.1177/1745691611406920 26168518

[ref11] Dienes Z : Using Bayes to get the most out of non-significant results. *Front. Psychol.* 2014;5:781. 10.3389/fpsyg.2014.00781 25120503 PMC4114196

[ref12] Dienes Z Mclatchie N : Four reasons to prefer Bayesian analyses over significance testing. *Psychon. Bull. Rev.* 2018;25(1):207–218. 10.3758/s13423-017-1266-z 28353065 PMC5862925

[ref13] Dopp AR Borduin CM Rothman DB : Evidence-Based Treatments for Youths Who Engage in Illegal Sexual Behaviors. *J. Clin. Child Adolesc. Psychol.* 2017;46(5):631–645. 10.1080/15374416.2016.1261714 28001446

[ref14] Dorsey S McLaughlin KA Kerns SEU : Evidence base update for psychosocial treatments for children and adolescents exposed to traumatic events. *J. Clin. Child Adolesc. Psychol.* 2017;46(3):303–330. 10.1080/15374416.2016.1220309 27759442 PMC5395332

[ref15] Evans SW Owens JS Bunford N : Evidence-based psychosocial treatments for children and adolescents with attention-deficit/hyperactivity disorder. *J. Clin. Child Adolesc. Psychol.* 2014;43(4):527–551. 10.1080/15374416.2013.850700 24245813 PMC4025987

[ref16] Fisher RA : Statistical methods for research workers. *Statistical Methods for Research Workers.* 1925.

[ref17] Freeman J Benito K Herren J : Evidence Base Update of Psychosocial Treatments for Pediatric Obsessive-Compulsive Disorder: Evaluating, Improving, and Transporting What Works. *J. Clin. Child Adolesc. Psychol.* 2018;47(5):669–698. 10.1080/15374416.2018.1496443 30130414

[ref18] Gallistel CR : The importance of proving the null. *Psychol. Rev.* 2009;116(2):439–453. 10.1037/a0015251 19348549 PMC2859953

[ref19] Glenn CR Esposito EC Porter AC : Evidence Base Update of Psychosocial Treatments for Self-Injurious Thoughts and Behaviors in Youth. *J. Clin. Child Adolesc. Psychol.* 2019;48(3):357–392. 10.1080/15374416.2019.1591281 31046461 PMC6534465

[ref20] Greenland S Senn SJ Rothman KJ : Statistical tests, P values, confidence intervals, and power: a guide to misinterpretations. *Eur. J. Epidemiol.* 2016;31(4):337–350. 10.1007/s10654-016-0149-3 27209009 PMC4877414

[ref21] Hogue A Henderson CE Becker SJ : Evidence Base on Outpatient Behavioral Treatments for Adolescent Substance Use, 2014-2017: Outcomes, Treatment Delivery, and Promising Horizons. *J. Clin. Child Adolesc. Psychol.* 2018;47(4):499–526. 10.1080/15374416.2018.1466307 29893607 PMC7192024

[ref22] Johnson VE : Properties of bayes factors based on test statistics. *Scand. J. Stat.* 2008;35(2):354–368. 10.1111/j.1467-9469.2007.00576.x

[ref23] Kaminski JW Claussen AH : Evidence base update for psychosocial treatments for disruptive behaviors in children. *J. Clin. Child Adolesc. Psychol.* 2017;46(4):477–499. 10.1080/15374416.2017.1310044 28459280 PMC5600477

[ref24] Kass RE Vaidyanathan SK : Approximate Bayes Factors and Orthogonal Parameters, with Application to Testing Equality of Two Binomial Proportions. *Journal of the Royal Statistical Society: Series B (Methodological).* 1992;54(1):129–144. 10.1111/j.2517-6161.1992.tb01868.x

[ref25] Kerr NL : HARKing: hypothesizing after the results are known. *Personal. Soc. Psychol. Rev.* 1998;2(3):196–217. 10.1207/s15327957pspr0203_4 15647155

[ref26] Kruschke JK Liddell TM : The Bayesian New Statistics: Hypothesis testing, estimation, meta-analysis, and power analysis from a Bayesian perspective. *Psychon. Bull. Rev.* 2018;25(1):178–206. 10.3758/s13423-016-1221-4 28176294

[ref27] Lin DY Zeng D : On the relative efficiency of using summary statistics versus individual-level data in meta-analysis. *Biometrika.* 2010;97(2):321–332. 10.1093/biomet/asq006 23049122 PMC3412575

[ref52] Mayo DG : *Statistical inference as severe testing: How to get beyond the statistics wars.* 1 ^st^ edition. Cambridge University Press;2018.

[ref28] Morey RD Romeijn J-W Rouder JN : The philosophy of Bayes factors and the quantification of statistical evidence. *J. Math. Psychol.* 2016;72:6–18. 10.1016/j.jmp.2015.11.001

[ref29] Morey RD and Rouder JN : BayesFactor: Computation of Bayes Factors for Common Designs. R package version 0.9.12-4.2. 2018. Reference Source

[ref30] Neyman J Pearson ES : On the Problem of the Most Efficient Tests of Statistical Hypotheses. *Philos. Trans. R. Soc. A Math. Phys. Eng. Sci.* 1933;231(694-706):289–337. 10.1098/rsta.1933.0009

[ref31] Nuijten MB Hartgerink CHJ Assen MALM van : The prevalence of statistical reporting errors in psychology (1985-2013). *Behav. Res. Methods.* 2016;48(4):1205–1226. 10.3758/s13428-015-0664-2 26497820 PMC5101263

[ref32] Oldehinkel AJ : Editorial: Bayesian benefits for child psychology and psychiatry researchers. *J. Child Psychol. Psychiatry.* 2016;57(9):985–987. 10.1111/jcpp.12619 27535649

[ref33] Pina AA Polo AJ Huey SJ : Evidence-Based Psychosocial Interventions for Ethnic Minority Youth: The 10-Year Update. *J. Clin. Child Adolesc. Psychol.* 2019;48(2):179–202. 10.1080/15374416.2019.1567350 30746965

[ref34] R Core Team, R: *R: A language and environment for statistical computing. [Computer software].* R Foundation for Statistical Computing;2020;

[ref35] Rouder JN Morey RD : Default Bayes Factors for Model Selection in Regression. *Multivar. Behav. Res.* 2012;47(6):877–903. 10.1080/00273171.2012.734737 26735007

[ref36] Rouder JN Speckman PL Sun D : Bayesian t tests for accepting and rejecting the null hypothesis. *Psychon. Bull. Rev.* 2009;16(2):225–237. 10.3758/PBR.16.2.225 19293088

[ref37] Sakaluk JK Williams AJ Kilshaw RE : Evaluating the evidential value of empirically supported psychological treatments (ESTs): A meta-scientific review. *J. Abnorm. Psychol.* 2019;128(6):500–509. 10.1037/abn0000421 31368729

[ref50] Schönbrodt FD : p-checker: One-for-all p-value analyzer. 2018. Reference Source

[ref38] Schönbrodt FD Wagenmakers E-J : Bayes factor design analysis: Planning for compelling evidence. *Psychon. Bull. Rev.* 2018;25(1):128–142. 10.3758/s13423-017-1230-y 28251595

[ref39] Shafer G : Lindley’s Paradox. *J. Am. Stat. Assoc.* 1982;77(378):325–334. 10.2307/2287244

[ref40] Shepard JA Poler JE Grabman JH : Evidence-Based Psychosocial Treatments for Pediatric Elimination Disorders. *J. Clin. Child Adolesc. Psychol.* 2017;46(6):767–797. 10.1080/15374416.2016.1247356 27911597

[ref41] Silverman WK Hinshaw SP : The second special issue on evidence-based psychosocial treatments for children and adolescents: A 10-year update. *J. Clin. Child Adolesc. Psychol.* 2008;37(1):1–7. 10.1080/15374410701817725 18444057

[ref42] Simmons JP Nelson LD Simonsohn U : False-positive psychology: undisclosed flexibility in data collection and analysis allows presenting anything as significant. *Psychol. Sci.* 2011;22(11):1359–1366. 10.1177/0956797611417632 22006061

[ref43] Szucs D Ioannidis JPA : When null hypothesis significance testing is unsuitable for research: A reassessment. *Front. Hum. Neurosci.* 2017;11:390. 10.3389/fnhum.2017.00390 28824397 PMC5540883

[ref44] Schoot R van de Winter SD Ryan O : A systematic review of Bayesian articles in psychology: The last 25 years. *Psychol. Methods.* 2017;22(2):217–239. 10.1037/met0000100 28594224

[ref45] Wagenmakers E-J Marsman M Jamil T : Bayesian inference for psychology. Part I: Theoretical advantages and practical ramifications. *Psychon. Bull. Rev.* 2018;25(1):35–57. 10.3758/s13423-017-1343-3 28779455 PMC5862936

[ref46] Weersing VR Jeffreys M Do M-CT : Evidence base update of psychosocial treatments for child and adolescent depression. *J. Clin. Child Adolesc. Psychol.* 2017;46(1):11–43. 10.1080/15374416.2016.1220310 27870579 PMC5296370

[ref47] Wetzels R Matzke D Lee MD : Statistical Evidence in Experimental Psychology: An Empirical Comparison Using 855 t Tests. *Perspectives on Psychological Science: A Journal of the Association for Psychological Science.* 2011;6(3):291–298. 10.1177/1745691611406923 26168519

[ref48] Williams AJ Botanov Y Kilshaw RE : Potentially harmful therapies: A meta-scientific review of evidential value. *Clin. Psychol. Sci. Pract.* 2020;28:5–18. 10.1111/cpsp.12331

