# Supplementary material for: Bayes factor benefits for clinical psychology: review of child and adolescent evidence base
Source: F1000Res. 2022 Sep 23;11:171. Originally published 2022 Feb 11. [Version 2] doi: 10.12688/f1000research.76842.2 (PMC10558984; doi:10.12688/f1000research.76842.2)
Supplement: Peer review history

# Reviewer report: Reviewer response for version 2

**DOI:** 10.5256/f1000research.138892.r151263

**Published:** 2023-10-06

**Authors:** Stephen Senn

**Affiliations:** 1 School of Health and Related Research, The University of Sheffield, Sheffield, UK

I do not consider that the authors have completely answered my criticisms. Unless I have misunderstood, they are only considering cases where there is a true effect. Thus, they cannot provide an estimate of false positive rates. That being so it is difficult to evaluate the performance of any method proposed. Some extremely bad methods would classify true positive studies as positive at an even higher rate.

 I am prepared to approve the study, however, on the grounds that what it has found and demonstrated may be of interest to those who wish to undertake a proper investigation of Bayes factors. Such an investigation would have to include the performance of Bayes factors under the null.

 I reject, however, the authors' conclusions. Bayes Factors may or may not "offer benefits for research in clinical psychology". However, this study does not demonstrate that this must be so.

Is the work clearly and accurately presented and does it cite the current literature?

Partly

If applicable, is the statistical analysis and its interpretation appropriate?

Partly

Are all the source data underlying the results available to ensure full reproducibility?

Yes

Is the study design appropriate and is the work technically sound?

Partly

Are the conclusions drawn adequately supported by the results?

No

Are sufficient details of methods and analysis provided to allow replication by others?

Partly

Reviewer Expertise:

Biostatistics and statistical methodology

I confirm that I have read this submission and believe that I have an appropriate level of expertise to confirm that it is of an acceptable scientific standard.

# Reviewer report: Reviewer response for version 1

**DOI:** 10.5256/f1000research.80824.r138920

**Published:** 2022-05-31

**Authors:** Stephen Senn

**Affiliations:** 1 School of Health and Related Research, The University of Sheffield, Sheffield, UK

The strangest thing about this study is that it claims the opposite of the usual Bayesian criticism of frequentist approaches. The usual criticism is that frequentist approaches give significance too easily. See, for example, ^1^^,^^2^^,^^3^. This immediately raises the question, "how have the authors come to their conclusion?". I am not sure what the answer is. I note, however, that the circumstances under which frequentist tests "give significance too easily", as noted by Casella and Berger ^4^, is where there is a lump of probability on H _0_. See Stephen Senn: The pathetic P-value (Guest Post) | Error Statistics Philosophy for a discussion and also ^5^.

 Now, in the simulation, it seems to me that the authors have simulated from the case where H _1_ is true. They state that they chose to "simulate 10,000 repeated studies based on the median observed effect size of the articles". But if the median observed effect size is not zero, then H _0_ is not true. However, the whole motivation of the proposal by Jeffreys of Bayes Factors in 1939 in his *Theory of Probability* was to be able to prove that scientific laws were true. This in turn required that a lump of probability should be placed on the null hypothesis. It is the fact that the probability under the alternative hypothesis is widely spread that causes some values under H _1_ to be less well supported (in terms of likelihood) than is the value under H _1_. It is this that leads to the Jeffreys-Lindley paradox ^6^^,^^7^. The authors mention this but don't seem to appreciate its relevance for their examination.  Thus, the simulation carried out by the authors does not reflect what Bayes factors were created to deal with.

 It is unclear to me whether this is the origin of the discrepancy. Nevertheless, it seems to me that the set-up the authors have used is confusing. To judge classification methods one needs a gold standard. This is usually provided by simulators by assumption and by varying such assumptions, the performance of two systems may be compared.

 In any case, to use either Bayes factors or P-values to make a decision to either accept or reject a hypothesis also requires the use of thresholds. Varying these will lead to different error rates. It is not clear to me that this aspect is properly addressed in this paper.

 I am also unhappy with the discussion of the history of statistics. P-values were in use before Fisher. For example, by Pearson ^8^ in 1900.  It was Fisher who gave them their modern interpretation, at least as early as 1925. The authors have Neyman and Pearson in 1933 improving on Fisher's approach of 1934, which would require a time machine.

Is the work clearly and accurately presented and does it cite the current literature?

Partly

If applicable, is the statistical analysis and its interpretation appropriate?

Partly

Are all the source data underlying the results available to ensure full reproducibility?

Yes

Is the study design appropriate and is the work technically sound?

Partly

Are the conclusions drawn adequately supported by the results?

No

Are sufficient details of methods and analysis provided to allow replication by others?

Partly

Reviewer Expertise:

Biostatistics and statistical methodology

I confirm that I have read this submission and believe that I have an appropriate level of expertise to confirm that it is of an acceptable scientific standard, however I have significant reservations, as outlined above.

## References

[1] : Revised standards for statistical evidence. *Proc Natl Acad Sci U S A* .2013;110(48) : 10.1073/pnas.1313476110 19313-7 10.1073/pnas.1313476110 24218581PMC3845140

[2] : An investigation of the false discovery rate and the misinterpretation of p-values. *R Soc Open Sci* .2014;1(3) : 10.1098/rsos.140216 140216 10.1098/rsos.140216 26064558PMC4448847

[3] : Redefine statistical significance. *Nat Hum Behav* .2(1) : 10.1038/s41562-017-0189-z 6-10 10.1038/s41562-017-0189-z 30980045

[4] : Reconciling Bayesian and Frequentist Evidence in the One-Sided Testing Problem. *Journal of the American Statistical Association* .1987;82(397) : 10.1080/01621459.1987.10478396 106-111 10.1080/01621459.1987.10478396

[5] : Two cheers for P-values?. *J Epidemiol Biostat* .2001;6(2) : 10.1080/135952201753172953 193-204; discussion 205 10.1080/135952201753172953 11434499

[6] : A STATISTICAL PARADOX. *Biometrika* .1957;44(1-2) : 10.1093/biomet/44.1-2.187 187-192 10.1093/biomet/44.1-2.187

[7] : A comment on D. V. Lindley's statistical paradox. *Biometrika* .1957;44(3-4) : 10.1093/biomet/44.3-4.533 533-534 10.1093/biomet/44.3-4.533

[8] : X.On the criterion that a given system of deviations from the probable in the case of a correlated system of variables is such that it can be reasonably supposed to have arisen from random sampling. *The London, Edinburgh, and Dublin Philosophical Magazine and Journal of Science* .1900;50(302) : 10.1080/14786440009463897 157-175 10.1080/14786440009463897

# Response

**Published:** 2022-09-20

**Authors:** Thomas Bertelsen

Thank you for your time and insightful remarks. We agree that our article may have been unclear on our goal, and especially in the discussion, we did not emphasize three critical aspects that you raised: 1. These findings are unexpected (opposite of usual critique) 2. The procedures are not applied in the conventional manner (we sample where H1 is assumed true) 3. All findings are contingent on thresholds (as are ours).

 In our new manuscript, we emphasize that our findings are unusual in the literature criticizing frequentism. The findings, however, are not surprising (at least in our profession), and it is crucial to highlight that clinical psychology research are often underpowered.

 We have also highlighted that our goal was not to see if Bayesian methods were more "right" than frequentist approaches, and that they were utilized in a particular case where we assumed that the initial findings were valid. Our goal was to see whether we could reach conclusions faster if the original study was correct. The paper's main point is that Bayesian approaches provide pragmatic advantages that are especially useful in clinical psychology. In the discussion, we sought to explain this point.

 In accordance with the aforementioned, we endeavored to emphasize that all conclusions are dependent on thresholds and that the benefits of Bayesian approaches are not better thresholds but rather more intuitive interpretation, which leads to more reasonable conclusions in the event of null results.

 We understand that our introduction to statistical methods is somewhat lacking. Our intent is that it should be readily available to researchers within the field of clinical psychology and therefore we wish to keep the background for the methods short. However, we have highlighted that this is an abridged history. If you would prefer, we could also include references to more in-depth discussions on the history of both Bayesian and frequentist methods.

 We appreciated your remark about time machines, and while Fisher caused innovation in many areas, I do not recall him being involved with time travel. The Fisher reference has been updated to the 1925 edition.

# Reviewer report: Reviewer response for version 1

**DOI:** 10.5256/f1000research.80824.r135924

**Published:** 2022-05-11

**Authors:** Einar Baldvin Baldursson

**Affiliations:** 1 Department of Communication and Psychology, Aalborg University, Aalborg, Denmark

I think that the researchers make a strong case for using Bayesian methods in clinical research. Few of the positives might profit from a bit stronger argumentation/illustration.

 The point that Bayesian methods lend themselves to at more adaptable research strategy is mentioned. This is actually a quite strong argument. Frequentist methods force a discipline on clinical research that makes such research more difficult to do.

 More doable research will enhance the pace of empirical testing and improving existing methods, and increase the likelihood of clinical research focusing on novel ideas. Bayesian methods will strengthen explorative and innovative clinical research.

 The burden of doing frequentist research is so heavy, that it primes researchers and clinicians to bet on "safe" and well established methods. In short - it is worth emphasizing that adapting Bayesian methods will increase innovation in clinical treatment and research.

 Secondly. And this point is implicit in the paper, but could be spelled out in a bit more words.

 Frequentist methods work wonderfully in a classical experimental setting where the focus is on a distinct part of a system, and the measurements are both fairly exact and reliable. None of this is possible in clinical research. The phenomena we observe are fussy, the measurements we use are inherently imprecise, and no amount of experimental control can master the dynamic processes and changes characteristic of phenomena in clinical research. Here Bayesian methods promise a better and more suitable approach.

 The final comment, also on something the authors mention. Frequentist methods increase the likelihood of false negatives. Bayesian methods decrease the likelihood of false negatives.

 The only real alternative to adopting the pragmatic approach of Bayesian methods, is to focus on more and more power. And that approach comes with a multitude of pitfalls.

 In short. In my view this paper makes an important contribution to the debate about how to approach and evaluate clinical research. 

 A bit stronger focus on the interaction between design and methods (the specific contribution of Bayesian methods) would strengthen the paper. On the other hand, maybe this part of the discussion merits a paper in its own right.

Is the work clearly and accurately presented and does it cite the current literature?

Yes

If applicable, is the statistical analysis and its interpretation appropriate?

Yes

Are all the source data underlying the results available to ensure full reproducibility?

Yes

Is the study design appropriate and is the work technically sound?

Yes

Are the conclusions drawn adequately supported by the results?

Yes

Are sufficient details of methods and analysis provided to allow replication by others?

Yes

Reviewer Expertise:

Research in occupational health, clinical research focusing on diagnosis and treatment in everyday life, and philosophy/method of science.

I confirm that I have read this submission and believe that I have an appropriate level of expertise to confirm that it is of an acceptable scientific standard.

# Response

**Published:** 2022-09-20

**Authors:** Thomas Bertelsen

Thank you for your time and encouragement. You have wonderfully captured the essence of this paper. In response to your concerns and those of Stephen Senn, we attempted to highlight the pragmatic merits of Bayesian methods.
